# The validity of the Type D construct and its assessment in Taiwan

**DOI:** 10.1186/1471-244X-13-46

**Published:** 2013-02-04

**Authors:** Chia-Ying Weng, Johan Denollet, Chin-Lon Lin, Tin-Kwang Lin, Wen-Chung Wang, Jyun-Ji Lin, Shu-Shu Wong, Floortje Mols

**Affiliations:** 1 Department of Psychology, National Chung-Cheng University, Chia-Yi, Taiwan; 2 Center of Research on Psychology in Somatic diseases (CoRPS), Tilburg University, Tilburg, The Netherlands; 3 Division of Cardiology, Department of Internal Medicine, The Buddhist Dalin Tzu Chi General Hospital, Chia-Yi, Taiwan; 4 School of Medicine, Tzu Chi University, Hualien, Taiwan; 5 The Hong Kong Institute of Education, Tai po, Hong Kong; 6 Department of Child Development and Family Studies, Tzu Chi University, Hualien, Taiwan

**Keywords:** Type D personality, DS14-T, Coronary artery disease, Taiwan

## Abstract

**Background:**

Type D (distressed) personality, defined by negative affectivity and social inhibition, is related to cardiovascular outcomes. Little is known about Type D in non-Western cultures. We examined the validity of this construct and its assessment in Taiwanese patients with coronary artery disease (CAD) and adults from the general population.

**Methods:**

CAD patients (*N* = 87) and adults from the general population (*N* = 421) completed the 14-item Type D Scale- Taiwanese version (DS14-T), State-Trait Anxiety Inventory, Beck Depression Inventory-II, and Chinese Hostility Inventory Short-Form.

**Results:**

Based on the psychometric examination, item #3 of the original DS14, "I often talk to strangers" was replaced by "I don't like to have a lot of people around me" which comes from the “Withdrawal” facet of social inhibition of DS-24. The reliability of Type D assessment in Taiwan was good, with Cronbach’s α for negative affectivity and social inhibition of .86 and .79. Factor analyses confirmed the two-factor model of the Type D construct. The prevalence rate of Type D personality in Taiwan was 20% in CAD patients and 16% in the general population. Negative affectivity was positively associated with anxiety, depression and hostility, and social inhibition was positively associated with suppressive hostility and negatively associated with expressive hostility after controlling for the total hostility. Furthermore, Taiwanese individuals with a Type D personality displayed elevated levels of anxiety, depression and hostility.

**Conclusions:**

The Type D construct and its assessment with the DS14-T is generalizable to an Asian setting, Taiwan. The DS14-T showed good psychometric properties, and the prevalence of Type D personality in Taiwan was similar to the prevalence rates in Western countries and Mainland China, and Type D was associated with anxiety, depression and hostility.

## Background

Type D personality has been shown to predict cardiac mortality in patients with coronary artery disease (CAD) [1,2], and other cardiac events in CAD and myocardial infarction patients [3-7]. Furthermore, Type D personality has also been shown to be a significant predictor of impaired quality of life in CAD patients [3,8-11]. Moreover, Type D personality was also associated with symptoms of anxiety and depression in patients who underwent cardiac surgery [12].

Type D personality refers to the combination of two global traits: negative affectivity (NA) and social inhibition (SI) [13]. People with high NA tend to experience negative emotions across time and situations [13-16]. They not only experience dysphoria and anxiety, but also hold negative attitudes toward themselves, report more physical symptoms, and have an attention bias toward negative stimuli [13], and experience lower levels of well-being than low NA individuals [17]. Denollet [14] indicated that high SI individuals often experience discomfort, nervousness, tension and insecurity when with others, but tend to inhibit their emotional and behavioral expression in social interaction to avoid the disapproval of others.

The Type D scale (DS14) was developed to assess NA, SI, and Type D personality [14] . The 7 NA items from the DS14 cover the tendency to experience feelings of dysphoria, anxiety, and irritability, and the 7 SI items cover social discomfort, reticence, and lack of social poise. The original DS14 has shown good psychometric properties (α = .88 and .86; 3-month test-retest reliability = .72 and .82 for NA and SI, respectively) [14]. Validation studies have shown that the NA correlated positively with anxiety [18-24], depression [18-24], perceived stress [20,21], and neuroticism [14,18-21,25], but correlated negatively with extraversion [14,18-21,25]. The SI correlated positively with anxiety [18-21,24], depression [18-21,24], and neuroticism [14,18-21,25], but correlated negatively with extraversion [14,18-21,25]. For samples of general population [14,19,21,25-27], cardiac patients [14,18,20-22,26,27], and other patients [14,21,23,27-29], the DS14 has demonstrated a stable two-factor structure and the scores have shown good psychometric properties. In addition, the DS14 has been used in epidemiologic and clinical research worldwide [14,19,20,22-25,27-29].

Type D personality has been studied extensively in Western countries such as Belgium (α_NA_ = .88; α_SI_ = .86) [14], Denmark (α_NA_ =.87; α_SI_ = .91) [18], Germany (α_NA_ = .87; α_SI_ = .86) [26], Iceland (α_NA_ = .85; α_SI_ = .84) [20], Israel (α_NA_ = .79; α_SI_ = .80) [23], Netherlands (α_NA_ = .88; α_SI_ = .86) [14], Norway (α_NA_ = .87; α_SI_ = .83) [22], Poland (α_NA_ = .86; α_SI_ = .84) [21], Turkey (α_NA_ = .82; α_SI_ = .81) [24], and Ukraine (α_NA_ = .86; α_SI_ = .71) [19]. However, there have been few studies in non-Western cultures such as China, and Korea [30-33]. Even though there are two DS14 Chinese versions developed in Mainland China [30,31], people in Taiwan cannot fully and easily understand the wording of them due to the cultural and wording differences between Mainland China and Taiwan, which come from the geographic separation along with the political and economical separations for some 50 years. Therefore, the purpose of the present study was to examine the validity of the Type D personality construct and its assessment with the DS14 in Taiwan. This study should enable researchers to compare the prevalence of the Type D personality and the prognostic power of Type D personality on cardiovascular diseases in Taiwan with other countries.

The definition of SI [14] includes multiple dimensions: (a) affect: discomfort, nervousness, and tension, which has been validated by previous studies [20,21]; (b) motivation: to avoid the disapproval of others, which was not supported by Grande [25] as his study failed to find a higher correlation between SI and impression management (other-deception) than that between SI and self-deception; (c) cognition: suspicious and insecurity beliefs toward interpersonal transactions; (d) behavior: inhibiting the emotional and behavioral expression in social interaction. In this study, we aimed to validate SI with the cognition and behavior dimensions and proposed the two following hypotheses. First, for the cognition dimension, SI is positively associated with hostile cognition. Second, for the behavior dimension, SI is positively associated with hostility suppression and negatively associated with hostility expression. Also, we hypothesized that NA is positively associated with anxiety, depression and hostility affect (anger) since Denollet [34] has proposed that Type D personality is a general propensity to psychological distress.

## Methods

### Participants and procedure

For this study, 421 adults from the general population and 87 CAD patients between 18 and 85 years of age (253 males and 255 females; mean age, 50.96 ± 14.89 years) were recruited from the Buddhist Dalin Tzu Chi General Hospital between October 2009 and October 2010. The general population was recruited from the outpatients of the health examination department, and the CAD patients were recruited from outpatients of the cardiology department. Exclusion criteria were life-threatening comorbid diseases, psychiatric disorders, and cognitive disability. All participants were informed about and invited to participate in the investigation by the study assistant at the clinic. About 30% of those invited refused to participate and no particularly systematic reasons were found for the refusal. All participants provided written informed consent. The Institutional Review Board (IRB) and the Research Committee of The Buddhist Dalin Tzu Chi General Hospital approved the study protocols. Instructions were also provided in writing. The assistant waited until each participant had answered all questionnaires. There was no time limit.

### Instruments

#### Assessment of Type D in Taiwan

The DS14 contains 14 items with a 5-point Likert Type scale (0 = false to 4 = true) that assess the 2 Type D components: 7 NA items (e.g., “I often make a fuss about unimportant things” and “I take a gloomy view of things”) to measure an individual’s disposition to experience negative emotions, and 7 SI items (e.g., “I make contact easily when I meet people” and “I find it hard to start a conversation”) to measure an individual’s disposition to inhibit the expression of emotions and behaviors during social interaction.

A forward–backward procedure was used to translate the original English-language version of the DS14 to form the first draft of DS14 in Taiwan which was then administered to Taiwanese CAD patients recruited from the Buddhist Dalin Tzu Chi General Hospital (*N* = 260) and general population recruited from the community (*N* = 84) between November 2006 and November 2007 by the similar recruitment procedure mentioned above. Exploratory factor analysis identified the two-factor structure of the scale, with NA accounting for 26.66% and SI 17.71% of the variance, respectively. The internal consistency was high for NA but not high enough for SI, with α of .83 and .67 for NA and SI, respectively [35]. Since α of SI was lower than .70 and the low item-total correlation (r = .18) for Item 3, “I often talk to strangers.” indicated that revision of the SI subscale was needed in Taiwan. Hence, we decided to replace Item 3 with a withdrawal item that belonged to the social inhibition subscale of an earlier 24-item version of the Type D scale [13], and modified the DS14 in order to increase the internal consistency of the SI assessment in Taiwan. Three additional items, “I would rather keep in the background”; “When I meet a lot of people, I get nervous”; and “I don't like to have a lot of people around me”, which reflect the ‘withdrawal’ facet of social inhibition [13] were considered to be candidate new items for the SI subscale of the DS14 in Taiwan. Because the item, “When I meet a lot of people, I get nervous” represents conceptually not only social inhibition but also negative affectivity dimension in Taiwan culture, only the other two items were kept with the original 7-item SI subscale to form the 9-item second draft of the SI subscale. Based on their psychometrical properties, we then chose the best seven items from the nine items to form the final 7-item SI subscale of the Taiwan version of the DS14 (DS14-T).

#### Anxiety, depression and hostility

Because Type D personality refers to a propensity towards general psychological distress, we examined the associations between Type D personality, anxiety, depression, and hostility. Anxiety was measured using the 20-item Chinese version of Trait Anxiety subscale of State-Trait Anxiety Inventory (STAI-TA) [36], which assessed an individual’s disposition to experience anxiety status. α was .93 for the Chinese version of STAI-TA [36]. Depression was measured using the 21-item Chinese Version of Beck Depression Inventory-II (BDI-II) [37], which assessed an individual’s levels of depressive symptoms. α was .94 for the Chinese version of BDI-II [37]. Hostility was measured using the 20-item Chinese Hostility Inventory Short-Form (CHI-SF) [38], which contained four dimensions of hostility: (1) hostile cognition (six items, α = .78); (2) hostile affection (four items, α = .78); (3) expressive hostility (five items, α = .76); and (4) suppressive hostility (five items, α = .73). α was .80 for the whole CHI-SF [38].

### Statistical analysis

The α coefficient was computed for internal consistency. An item would be deleted if both its item-total correlation was smaller than .30 and such a deletion would lead to an increase in α. The literature [30,31] has shown that the factor structure of the DS14 was very similar between the CAD patients and the general population. Thus, all participants were merged together. To evaluate invariance of the scale and model-data fit, we randomly divided the participants into two halves and conducted exploratory factor analysis (EFA) and confirmatory factor analysis (CFA) on each half, respectively. In the EFA, a principal axis factoring analysis with oblique rotation (direct oblimin method) was adopted. In the CFA, the AMOS statistical software package Version 18 [39] was adopted. Several popular fit indices were used to evaluate model-data fit, including the chi-square fit index (*χ*^*2*^), the ratio of chi-square to the degree of freedom (*χ*^2^/*df*), the standardized root mean square residual (SRMR), the root mean square error of approximation (RMSEA), Akaike information criterion (AIC), the comparative fit index (CFI), the incremental fit index (IFI), the goodness-of-fit-index (GFI), and the adjusted GFI (AGFI).

Pearson’s correlations were computed to estimate the criterion-related validity of the scale against anxiety, depression, and hostility. The partial correlations were calculated to examine the relationships between suppressive hostility and expressive hostility with SI after controlling for the total hostility score. The independent t-test was used to examine the differences in mean levels of anxiety, depression and hostility between Type D and non-Type D individuals. All data were analyzed by SPSS 18.0 and AMOS 18.0.

## Results

The results of the study fall into five main areas: (1) reliability estimates for the scores on the NA and SI subscales; (2) factor analysis of the hypothesized factor structure; (3) convergent and divergent validity for the correlations between NA and SI with other psychological factors; (4) prevalence of Type D personality in Taiwan; and (5) differences in anxiety, depression and hostility between the Type D vs. non -Type D individuals. The demographic characteristics of the participants are presented in Table 1. The characteristics of the participants (gender, age, and marital status) were similar to those in the Chinese study [30].

**Table 1:** Demographic characteristics of the study population

	**CAD patients**		**General participants**	
	**(*N *= 87)**		**(*N *= 421)**	
**Characteristics**	***n***	**%**	***n***	**%**
Gender				
Male	66	75.86	187	44.42
Female	21	24.14	234	55.58
Age groups(years)				
≦ 49	12	13.79	212	50.36
50-59	27	31.03	103	24.47
60-69	27	31.03	70	16.63
≧ 70	21	24.14	36	8.55
Marital status^a^				
Married	73	86.90	317	76.20
Single/Divorced/Separated/Widowed	11	13.10	99	23.80

^a^3 CAD patients and 5 general participants did not report their marital status.

### Reliability

Table 2 presents the reliability analysis of the NA and SI subscales for the DS14-T. The results showed two of the nine items in the SI subscale, “I often talk to strangers” (Item #3) and “I would rather keep in the background” (Item #15), should be deleted. Based on the psychometrical analysis, Item #3 was replaced by “I don't like to have a lot of people around me” (Item #16) in the final SI subscale of DS14-T. No items in the NA subscale were deleted. Finally, there were seven items in each subscale of DS14-T. Cronbach’s α was.86 and .79 for the NA and SI subscales, respectively, indicating good internal consistency [40].

**Table 2:** **Item-total correlation and Cronbach's alpha with item deleted of Type D Scale-14 Taiwanese version (*N *= 508)**

	**Item-Total Correlation**			**α if Item Deleted**		
**Item Content**	**DS14**	**DS16-T**	**DS14-T**	**DS14**	**DS16-T**	**DS14-T**
Negative affectivity				.86^a^	.86 ^a^	.86 ^a^
2. I often make a fuss about unimportant things.	.44	.44	.44	.87	.87	.87
4. I often feel unhappy.	.69	.69	.69	.83	.83	.83
5. I am often irritated.	.73	.73	.73	.83	.83	.83
7. I take a gloomy view of things.	.61	.61	.61	.84	.84	.84
9. I am often in a bad mood.	.72	.72	.72	.83	.83	.83
12. I often find myself worrying about something.	.58	.58	.58	.85	.85	.85
13. I am often down in the dumps.	.69	.69	.69	.83	.83	.83
Social inhibition				.76 ^a^	.76 ^a^	.79 ^a^
1. I make contact easily when I meet people.	.47	.42	.39	.74	.74	.79
**3. I often talk to strangers.**	**.30**	**.30**	**-**	**.78**	**.77**	**-**
6. I often feel inhibited in social interactions.	.54	.53	.60	.72	.73	.75
8. I find it hard to start a conversation.	.59	.57	.59	.71	.72	.75
10. I am a closed kind of person.	.51	.52	.55	.73	.73	.76
11. I would rather keep other people at a distance.	.45	.50	.50	.74	.73	.77
14. When socializing, I don’t find the right things to talk about.	.57	.58	.58	.72	.72	.75
**15. I would rather keep in the background.**	**-**	**.21**	**-**		**.78**	**-**
16. I don't like to have a lot of people around me.	-	.46	.45		.74	.78

DS14 = Original 14-items Type D Scale; DS16-T = Added 2 new items in DS14 in Taiwan; DS14-T = final 14-item Taiwan version DS14;

α = Cronbach's alpha coefficient; items with the item-total correlation smaller than .30 and an increase in Cronbach’s alpha when deleted are in boldface.

^a^ Cronbach's alpha coefficient of subscale.

### Factor analyses

#### Exploratory factor analysis (EFA)

Table 3 presents the EFA analysis of DS14-T. The results showed that appropriateness of factor analysis was supported by Bartlett’s test of sphericity, *χ*^*2*^(91) = 1368.62, *p* < .001, and the Kaiser-Meyer-Olkin measurement of sampling adequacy index, .91, indicating that the sample and correlation matrix were appropriate for factor analysis. Multiple methods including Kaiser’s eigenvalue >1 criterion, Cattell’s scree test, and parallel analysis were used to determine the number of factors and the results showed that a two-factor solution was the best.

**Table 3:** **Factor loadings for exploratory factor analysis with direct oblimin rotation of Type D Scale 14-Taiwanese version (*N *= 254)**

		**DS14**			**DS16-T**			**DS14-T**				
		**NA**	**SI**	***h***^***2***^	**NA**	**SI**	***h***^***2***^	**NA**	**SI**	***h***^***2***^	***M***	***SD***
Factor 1: Negative Affectivity												
2.	I often make a fuss about unimportant things.	**.52**	-.12	.24	**.53**	-.12	.25	**.53**	-.10	.23	1.10	1.27
4.	I often feel unhappy.	**.70**	.07	.53	**.70**	.07	.53	**.71**	.06	.55	0.99	1.29
5.	I am often irritated.	**.82**	-.08	.62	**.80**	-.04	.62	**.85**	-.08	.65	1.33	1.24
7.	I take a gloomy view of things.	**.65**	.12	.49	**.63**	.13	.48	**.55**	.21	.47	0.88	1.18
9.	I am often in a bad mood.	**.77**	.12	.68	**.76**	.13	.68	**.75**	.14	.68	1.10	1.28
12.	I often find myself worrying about something.	**.62**	.08	.42	**.59**	.13	.42	**.59**	.10	.43	0.85	1.10
13.	I am often down in the dumps.	**.71**	.09	.57	**.70**	.11	.57	**.68**	.13	.57	1.20	1.28
Factor 2: Social Inhibition												
1.	I make contact easily when I meet people.	-.07	**.52**	.25	-.07	**.46**	.19	-.08	**.41**	.14	1.14	1.37
3.	I often talk to strangers.	-.16	**.44**	.17	-.18	**.45**	.17	-	-	-	-	-
6.	I often feel inhibited in social interactions.	.24	**.55**	.45	.21	**.55**	.44	.06	**.67**	.49	0.70	1.05
8.	I find it hard to start a conversation.	.24	**.59**	.51	.20	**.59**	.48	.13	**.59**	.45	1.76	1.43
10.	I am a closed kind of person.	.25	**.49**	.39	.21	**.52**	.40	.04	**.65**	.46	1.20	1.30
11.	I would rather keep other people at a distance.	.25	**.44**	.33	.19	**.50**	.37	.09	**.56**	.37	0.75	1.11
14.	When socializing, I don’t find the right things to talk about.	.13	**.61**	.45	.07	**.66**	.48	-.05	**.71**	.47	0.81	1.14
15.	I would rather keep in the background.	-	-	-	.03	.27	.08	-	-	-	-	-
16.	I don't like to have a lot of people around me.	-	-	-	.23	**.43**	.32	.17	**.45**	.31	1.27	1.29

DS14 = Original 14-items Type D Scale; DS16-T = Added 2 new items in DS14 in Taiwan; DS14-T = final 14-item Taiwan version DS14;

Factor loadings > .40 are in boldface; NA = Negative affectivity; SI = Social Inhibition; *h*^*2*^ = communalities of the measured variables.

Because the purpose of EFA was to identify the most plausible model with meaningful factors underlying the scale, two criteria were used to determine the factor structure: (a) retaining items with factor loadings exceeding .40; and (b) retaining factors with at least three items. The two-factor solution provided the clearest factor structure, and no items were deleted. The two-factor solution was not only statistically sound but it also maintained the theoretical integrity of the model.

The correlation between the two factors was .54. Table 3 presents the two factors with their respective items: factor loadings, communality estimates (*h*^*2*^), means, and standard deviations. According to the factor pattern coefficients, each item loaded on its corresponding factor, with the coefficient ranging from .41 to .85 and communality value ranging from .14 to .68. The first factor, consisting of seven items, was named “Negative Affectivity” because the items reflected the tendency to experience feelings of dysphoria, anxiety, and irritability. The second factor, consisting of seven items, was named “Social Inhibition” because the items reflected social discomfort, reticence, and lack of social poise.

#### Confirmatory factor analysis (CFA)

A CFA was conducted to directly test the hypothesized two-factor structure of the DS14. Two models were tested. Model 1 examined the structure of the DS14-T as indicated by Denollet [14]. It contained two correlated factors, NA and SI, each factor with seven unique items. Model 2 posited a single factor on the 14 items. Multiple indices provided a comprehensive evaluation of model fit. Table 4 showed the two-factor model had a better fit than the one-factor model, primarily because it had the following lowest statistics: *χ*^*2*^*, χ*^2^/*df,* AIC, SRMR, and RMSEA. Using the likelihood ratio test, the chi-square difference statistics for the two-factor model versus the one-factor model was significant (*p* < .001). In addition, results also suggested that the two-factor model was a good fit to the data, as indicated by the *χ*^2^/*df* (2.24) which was slightly greater than 2; the SRMR (.07) less than .08; the RMSEA (.07) was close to .06; the GFI (.91), CFI (.92) and IFI (.92) were greater than .90; and the AGFI (.88) was close to .90. All the fit indices suggested a reasonable model fit.

**Table 4:** **Goodness of fit indices for two-factor and one-factor models (*N *= 254)**

	**Two-factor model**		**One-factor model**	
*χ*^*2*^	170.04		432.04	
*df*	76		77	
*χ*^2^/*df*	2.24		5.64	
SRMR	.07		.12	
RMSEA	.07		.14	
AIC	228.04		490.04	
CFI	.92		.71	
IFI	.92		.71	
GFI	.91		.74	
AGFI	.88		.64	

*χ*^*2*^ = chi-square fit index; *χ*^2^/*df* = the ratio of chi-square to the degree of freedom; SRMR = standardized root mean square residual; RMSEA = root mean square error of approximation; AIC = Akaike information criterion; CFI = comparative fit index; IFI = incremental fit index; GFI = goodness-of-fit index; AGFI = adjusted GFI.

### Convergent and divergent validity

As hypothesized, results showed a positive correlation of NA with anxiety (.67), depression (.50) and hostile affect (.55)(Table 5). Furthermore, the correlation between hostile cognition and SI was .34. Moreover, the partial correlations between suppressive hostility and expressive hostility with SI were .25 and -.19 after controlling for the total hostility score. All statistical significances reached the .001 level. The results indicated that the construct of Type D personality was consistent with the theoretical expectations.

**Table 5:** Correlations of negative affectivity and social inhibition to psychological factors

**Variables**	**1**	**2**	**3**	**4**	**5**	**6**	**7**	**8**
1. Anxiety	-							
2. Depression	.62^*^	-						
3. Hostile cognition	.42^*^	.34^*^	-					
4. Hostile affect	.49^*^	.32^*^	.49^*^	-				
5. Expressive hostility	.33^*^	.25^*^	.46^*^	.47^*^	-			
6. Suppressive hostility	.51^*^	.42^*^	.56^*^	.50^*^	.33^*^	-		
7. Total hostility score	.56^*^	.44^*^	.83^*^	.76^*^	.72^*^	.79^*^	-	
8. Negative affectivity	.67^*^	.50^*^	.52^*^	.55^*^	.35^*^	.53^*^	.63^*^	-
9. Social inhibition	.40^*^	.25^*^	.34^*^	.33^*^	.20^*^	.48^*^	.45^*^	.47^*^

^*^P <.001.

### Prevalence of Type D personality in the Taiwanese population

The median for the NA and SI subscales was 6 and 6, respectively. For international comparability, we used this standard cut-off score (both NA≧10 and SI≧10) to calculate the prevalence: 20% of the CAD patients and 16% of the general population were defined as having a Type D personality. Although the present study was not an epidemiological study, it is noteworthy that the estimates fall within the range of what has been reported in Western countries as well as Mainland China [30,31].

### Differences in anxiety, depression and hostility between the Type D vs. non -Type D individuals

Individuals with a Type D personality showed a significantly (all *P*’s <0.001) higher score on the anxiety, depression, hostile cognition, hostile affect, expressive hostility, suppressive hostility, and total hostility score in comparison to those without a Type D personality (Table 6).

**Table 6:** Mean scores on the measures compared between Type D and non-Type D

	**Type D**			**Non-Type D**			**t**	**Cohen’s d**
**Variables**	***N***	***M***	***SD***	***N***	***M***	***SD***		
1. Anxiety	79	45.59	8.22	396	34.26	8.85	10.52^*^	1.33
2. Depression	80	7.46	6.92	399	3.70	4.46	4.67^*^	0.65
3. Hostile cognition	83	16.66	4.34	416	12.29	5.00	7.43^*^	0.93
4. Hostile affect	85	10.51	3.47	414	7.04	3.14	9.11^*^	1.05
5. Expressive hostility	84	12.15	4.14	412	9.77	4.18	4.78^*^	0.57
6. Suppressive hostility	84	16.51	3.79	416	11.66	4.54	10.32^*^	1.16
7. Total hostility score	82	55.89	10.88	408	40.77	12.89	11.12^*^	1.27

^*^P < .001.

## Discussion

The purpose of the present study was to examine the validity of the Type D personality construct and its assessment with the DS14 in Taiwan. α was .86 and .79 for the NA and SI subscales, respectively, suggesting good reliability [40]. In additon, it was higher than that in the previous Taiwanese version (α was .83 and .67 the NA and SI subscales, respectively) [35]. Furthermore, EFA and CFA supported the two-factor structure of the scale. The results of criterion-related validity were consistent with previous research in positive correlations between NA with anxiety [17,41,42], depression [17,41], and hostility [43,44]. The present study expanded the previous research to validate that SI is positively correlated with hostility suppression while negatively correlated with hostility expression. These findings further validate the construct of Type D personality and confirm that Type D personality is generalizable in an Asian setting such as Taiwan.

In the present study, Item 3, "I often talk to strangers" was removed from the SI subscale of the DS14 because of the low item-total correlation (.30) and the increase in alpha when the item was deleted. This result is consistent with two of the modification works conducted in Asian area [31,32]. Bai et al.’s study [31], the modification work of the DS14 in Mainland China, suggested the re-modification of the DS14 because of the low factor loading (.34) and the lack of discrimination power of item 3. Of the 146 participants in their study, 71.9% answered this item as false or rather false. One possible explanation proposed by that study for the failure of item 3 to assess the social inhibition in Mainland China may be social customs which are different from those of the Western culture. We agree with this explanation because in both Chinese and Taiwanese societies, especially in conservative rural areas, people are not accustomed to talking to strangers. That may be the reason item 3 performed poorly in Taiwan and Mainland China [30,31] and did perform well in Hong Kong [33] from a psychometric point of view. Furthermore, Lim et al.’s study [32], the modification work of the DS14 in Korea, also replaced Item 3 with new item basing on psychometric grounds. Since Item 3, "I often talk to strangers" may not be suitable for use in all of the Asian culture, especially not for conservative rural areas, it was replaced by the new item: "I don't like to have a lot of people around me" to form the formal seven-item SI subscale of the DS14-Taiwanese version.

Suls and Bunde [45] pointed out that negative affective dispositions such as hostility, depression and anxiety may overlap with each other. The correlation results of the present study supported the overlap among Type D personality, anxiety, depression and hostility, which is consistent with the previous findings [34,44].

Meta-analyses showed that both Type D personality [1] and hostility [46] are related to the prognosis of CAD. Furthermore, hostility is associated with autonomic nervous system imbalance [47]. Recently, we reported on the association between Type D personality and autonomic nervous system imbalance with a sample of 51 (age 51.4 ± 8.08, female: 49%) healthy adults who were free from disease and medication use [48]. For these reasons, we chose hostility, as assessed with the Chinese Hostility Inventory, as a validity base for the Type D construct.

According to the hypothetical statement proposed by Denollet [14] that high SI individuals not only experience more negative feelings, but they also tend to inhibit their emotions and behavior expression of these emotions in interpersonal situations [14,49], we hypothesized that, for the cognitive dimension, high SI individuals would have more hostile cognition and perceive their environment as unfriendly and even threatening. On the behavior dimension, they would tend to adopt more suppressive behaviors than expressive ones in interpersonal situations. The results of the present study support our expectations. SI was positively associated with hostile cognition. Furthermore, after controlling for the total hostility score, SI was positively associated with the suppressive hostility , at the meanwhile, negatively associated with expressive hostility.

Because expressive and suppressive behaviors are associated with different emotional and physical response patterns, as well as different behavioral and social consequences [24], further research is needed to explore the mechanisms underlying the possible linkage between suppressive behavior and the prognosis of CAD. Also, it should be noted that empirical study found that Asian Americans reported a higher level of habitual suppression than did Caucasians [50]. Thus, social inhibition may be more culturally acceptable in Asian settings than in Western settings. Further research is therefore warranted to test whether Type D personality is also a prognostic factor of CAD in Taiwan.

It is noteworthy that in this new Taiwanese version of the DS14, in comparison with the existing Chinese and Western versions, the validation base is broadened to cover the previously missed criteria of cognitive and behavioral dimensions of social inhibition. This strengthening of validation is particularly relevant in establishing the construct validity of social inhibition.

Some limitations of this study should be mentioned. A well-known personality measure such as the Big Five could be a better validating base for Type D personality. But, to our knowledge, there is no such questionnaire with consistent factor structure available in Chinese or Taiwanese that can be used to validate the negative affectivity construct [51,52]. Therefore, we used the Chinese Version of Beck Depression Inventory-II to measure depressive mood. Furthermore, self-rated instrument was used to obtain the evidence for the criterion-related validity in this study. Future research using direct behavior observation techniques is needed to provide more objective validity evidences.

## Conclusions

In conclusion, the Type D construct and its assessment with the DS14 is generalizable to an Asian setting, Taiwan. The DS14 showed good psychometric properties, the prevalence of Type D personality in Taiwan was similar to prevalence rates in Western countries and Mainland China, and Type D was associated with anxiety, depression and hostility.

## Competing interests

The authors declare that they have no competing interests.

## Authors’ contributions

All authors contributed to different aspects of this study. But the role of each author is equally important. WCY designed the study and drafted all the manuscript. Several physicians (LCL and LTK) contributed to conceptualization and design, the recruitment of participants, and the interpretation of data. JD and FM provided new items, reviewed and revised the manuscript critically for the theoretical construction. The rest of the authors (WWC, LJJ, and WSS) contributed to statistical analysis, interpretation of data and revision of the manuscript. All authors read and approved the final manuscript.

## Pre-publication history

The pre-publication history for this paper can be accessed here:

http://www.biomedcentral.com/1471-244X/13/46/prepub
